# Supplementary material for: Situational and Dispositional Achievement Goals and Measures of Sport Performance: A Systematic Review with a Meta-Analysis
Source: Sports (Basel). 2024 Nov 4;12(11):299. doi: 10.3390/sports12110299 (PMC11598045; doi:10.3390/sports12110299)
Supplement: Supplementary file 1 [file sports-12-00299-s001.zip › sports-3187798-supplementary.pdf]

Supplemental Tables and Figures

Contents:

- **Supplemental Table S1.** Study quality ratings.
- **Supplement Table S2.** CMA data file.
- **Supplemental Figures.** Funnel plots.
- **Supplement Figures.** Remove-one study figures.

Supplemental Table S1. Study quality ratings.

Study / Question	1	2	3	4	5	6	7	8	9	10	11	12	13	14
Alverez et al. [49]	2	2	2	2	N/A	N/A	N/A	2	N/A	2	2	N/A	2	2
Balaguer et al. [50]	2	2	1	2	N/A	N/A	N/A	2	N/A	2	2	N/A	2	2
Balaguer et al. [51]	2	2	1	2	N/A	N/A	N/A	2	N/A	2	2	N/A	2	2
Bono & Livi [52]	2	2	1	2	N/A	N/A	N/A	2	N/A	2	2	N/A	2	2
Bortoli et al. [53]	2	2	1	2	N/A	N/A	N/A	2	N/A	2	2	N/A	2	2
Boyd & Callaghan [54]	2	2	1	2	N/A	N/A	N/A	2	N/A	2	2	N/A	2	2
Branco et al. [55]	2	2	1	2	N/A	N/A	N/A	2	N/A	2	2	N/A	2	2
Cervello et al. [56]	2	2	1	2	N/A	N/A	N/A	2	N/A	2	2	N/A	2	2
Cumming et al. [57]	2	2	2	2	N/A	N/A	N/A	2	N/A	2	2	N/A	2	2
Dewar & Kavussanu [58]	2	2	2	2	N/A	N/A	N/A	2	N/A	2	2	N/A	2	2
e Silva et al. [59]	2	2	2	2	N/A	N/A	N/A	2	N/A	2	2	N/A	2	2
Elferink-Gemser et al. [60]	2	2	1	2	N/A	N/A	N/A	2	N/A	2	2	N/A	2	2
Farkhondeh & Moghaddam [61]	1	2	2	1	N/A	N/A	N/A	2	N/A	1	0	N/A	1	1
Figueiredo et al. [62]	2	2	2	2	N/A	N/A	N/A	2	N/A	2	2	N/A	2	2
García-Calvo et al. [63]	2	2	2	2	N/A	N/A	N/A	2	N/A	2	2	N/A	2	2
Holgado et al. [64]	2	2	0	2	N/A	N/A	N/A	2	N/A	2	0	N/A	2	2
Höner & Feichtinger [65]	2	2	2	2	N/A	N/A	N/A	2	N/A	1	2	N/A	2	2
Huijgen et al. [66]	2	2	1	2	N/A	N/A	N/A	2	N/A	2	2	N/A	2	2
Jeong [67]	2	2	2	2	N/A	N/A	N/A	2	N/A	2	1	N/A	2	2
Kim [68]	2	2	2	2	N/A	N/A	N/A	2	N/A	2	2	N/A	2	1
Knoblochova et al. [69]	2	2	1	2	N/A	N/A	N/A	2	N/A	2	2	N/A	1	2

Lemyre et al. [70]	2	2	2	2	N/A	N/A	N/A	2	N/A	2	2	N/A	2	2
Peng & Zhang [71]	2	2	2	2	2	0	0	2	2	2	2	2	2	2
Santos-Rosa et al. [72]	2	2	1	2	N/A	N/A	N/A	2	N/A	2	2	N/A	2	2
Smith et al. [73]	2	2	1	2	N/A	N/A	N/A	2	N/A	2	2	N/A	2	2
Tello et al. [74]	2	2	2	2	N/A	N/A	N/A	2	N/A	2	0	N/A	2	2
Tenenbaum et al. [75]	1	2	0	2	2	0	N/A	2	1	2	1	1	2	2
van de Pol & Kavussanu [76]	2	2	2	2	N/A	N/A	N/A	2	N/A	2	2	N/A	2	2
Van-Yperen & Duda [77]	2	2	1	2	N/A	N/A	N/A	2	N/A	2	2	N/A	2	2
Vazou [78]	2	2	1	2	N/A	N/A	N/A	2	N/A	2	2	N/A	2	2

Supplement Table S2. CMA data file.

	Study name	Subgroup	Data format	Correlation	Sample size	Effect direction	Correlation	Std Err	Fisher's Z	Std Err	Data type	Variable	Moderator Goal by Sit or Dis	Subjective or Objective	Performance viewpoint	Performance target	Athlete level	Elite vs. Non-Elite
1	Alvarez et al. [49]	EOP1	Corr, N	0.01	155	1	0.01	8.11E-02	1.00E-02	8.11E-02	Corr, N	Ego Orientation	Ego Orientation	Subjective	Athlete	Athlete	Mix	Non-Elite
2	Alvarez et al. [49]	EOP2	Corr, N	0.17	155	1	0.17	7.88E-02	0.171667	8.11E-02	Corr, N	Ego Orientation	Ego Orientation	Subjective	Athlete	Athlete	Mix	Non-Elite
3	Alvarez et al. [49]	TOP1	Corr, N	0.03	155	1	0.03	8.10E-02	3.00E-02	8.11E-02	Corr, N	Task Orientation	Task Orientation	Subjective	Athlete	Athlete	Mix	Non-Elite
4	Alvarez et al. [49]	TOP2	Corr, N	0.13	155	1	0.13	7.97E-02	0.13074	8.11E-02	Corr, N	Task Orientation	Task Orientation	Subjective	Athlete	Athlete	Mix	Non-Elite
5	Balaguer et al. [50]	ECP1	Corr, N	-0.04	181	1	-0.04	7.48E-02	-4.00E-02	7.50E-02	Corr, N	Ego Climate	Ego Climate	Subjective	Athlete	Athlete	Elite	Elite
6	Balaguer et al. [50]	ECP10	Corr, N	0.12	181	1	0.12	7.39E-02	0.120581	7.50E-02	Corr, N	Ego Climate	Ego Climate	Subjective	Athlete	Team	Elite	Elite
7	Balaguer et al. [50]	ECP11	Corr, N	-0.04	181	1	-0.04	7.48E-02	-4.00E-02	7.50E-02	Corr, N	Ego Climate	Ego Climate	Subjective	Athlete	Team	Elite	Elite
8	Balaguer et al. [50]	ECP12	Corr, N	0.11	181	1	0.11	7.40E-02	0.110447	7.50E-02	Corr, N	Ego Climate	Ego Climate	Subjective	Athlete	Team	Elite	Elite
9	Balaguer et al. [50]	ECP13	Corr, N	0.11	181	1	0.11	7.40E-02	0.110447	7.50E-02	Corr, N	Ego Climate	Ego Climate	Subjective	Athlete	Team	Elite	Elite

10	Balaguer et al. [50]	ECP14	Corr, N	0.2	181	1	0.2	7.20E-02	0.202733	7.50E-02	Corr, N	Ego Climate	Ego Climate	Subjective	Athlete	Team	Elite	Elite
11	Balaguer et al. [50]	ECP2	Corr, N	-0.09	181	1	-0.09	7.43E-02	-9.02E-02	7.50E-02	Corr, N	Ego Climate	Ego Climate	Subjective	Athlete	Athlete	Elite	Elite
12	Balaguer et al. [50]	ECP3	Corr, N	0.03	181	1	0.03	7.49E-02	3.00E-02	7.50E-02	Corr, N	Ego Climate	Ego Climate	Subjective	Athlete	Athlete	Elite	Elite
13	Balaguer et al. [50]	ECP4	Corr, N	-0.08	181	1	-0.08	7.45E-02	-8.02E-02	7.50E-02	Corr, N	Ego Climate	Ego Climate	Subjective	Athlete	Athlete	Elite	Elite
14	Balaguer et al. [50]	ECP5	Corr, N	-0.07	181	1	-0.07	7.46E-02	-7.01E-02	7.50E-02	Corr, N	Ego Climate	Ego Climate	Subjective	Athlete	Athlete	Elite	Elite
15	Balaguer et al. [50]	ECP6	Corr, N	0.11	181	1	0.11	7.40E-02	0.110447	7.50E-02	Corr, N	Ego Climate	Ego Climate	Subjective	Athlete	Athlete	Elite	Elite
16	Balaguer et al. [50]	ECP7	Corr, N	0.07	181	1	0.07	7.46E-02	7.01E-02	7.50E-02	Corr, N	Ego Climate	Ego Climate	Subjective	Athlete	Athlete	Elite	Elite
17	Balaguer et al. [50]	ECP8	Corr, N	0.07	181	1	0.07	7.46E-02	7.01E-02	7.50E-02	Corr, N	Ego Climate	Ego Climate	Subjective	Athlete	Team	Elite	Elite
18	Balaguer et al. [50]	ECP9	Corr, N	-0.02	181	1	-0.02	7.49E-02	-2.00E-02	7.50E-02	Corr, N	Ego Climate	Ego Climate	Subjective	Athlete	Team	Elite	Elite
19	Balaguer et al. [50]	EOP1	Corr, N	0.08	181	1	0.08	7.45E-02	8.02E-02	7.50E-02	Corr, N	Ego Orientation	Ego Orientation	Subjective	Athlete	Athlete	Elite	Elite
20	Balaguer et al. [50]	EOP10	Corr, N	-0.01	181	1	-0.01	7.49E-02	-1.00E-02	7.50E-02	Corr, N	Ego Orientation	Ego Orientation	Subjective	Athlete	Team	Elite	Elite
21	Balaguer et al. [50]	EOP11	Corr, N	-0.11	181	1	-0.11	7.40E-02	-0.11045	7.50E-02	Corr, N	Ego Orientation	Ego Orientation	Subjective	Athlete	Team	Elite	Elite
22	Balaguer et al. [50]	EOP12	Corr, N	-0.07	181	1	-0.07	7.46E-02	-7.01E-02	7.50E-02	Corr, N	Ego Orientation	Ego Orientation	Subjective	Athlete	Team	Elite	Elite
23	Balaguer et al. [50]	EOP13	Corr, N	-0.04	181	1	-0.04	7.48E-02	-4.00E-02	7.50E-02	Corr, N	Ego Orientation	Ego Orientation	Subjective	Athlete	Team	Elite	Elite

24	Balaguer et al. [50]	EOP14	Corr, N	0.02	181	1	0.02	7.49E-02	2.00E-02	7.50E-02	Corr, N	Ego Orientation	Ego Orientation	Subjective	Athlete	Team	Elite	Elite
25	Balaguer et al. [50]	EOP2	Corr, N	0.03	181	1	0.03	7.49E-02	3.00E-02	7.50E-02	Corr, N	Ego Orientation	Ego Orientation	Subjective	Athlete	Athlete	Elite	Elite
26	Balaguer et al. [50]	EOP3	Corr, N	0.07	181	1	0.07	7.46E-02	7.01E-02	7.50E-02	Corr, N	Ego Orientation	Ego Orientation	Subjective	Athlete	Athlete	Elite	Elite
27	Balaguer et al. [50]	EOP4	Corr, N	-0.01	181	1	-0.01	7.49E-02	-1.00E-02	7.50E-02	Corr, N	Ego Orientation	Ego Orientation	Subjective	Athlete	Athlete	Elite	Elite
28	Balaguer et al. [50]	EOP5	Corr, N	0.07	181	1	0.07	7.46E-02	7.01E-02	7.50E-02	Corr, N	Ego Orientation	Ego Orientation	Subjective	Athlete	Athlete	Elite	Elite
29	Balaguer et al. [50]	EOP6	Corr, N	0.11	181	1	0.11	7.40E-02	0.110447	7.50E-02	Corr, N	Ego Orientation	Ego Orientation	Subjective	Athlete	Athlete	Elite	Elite
30	Balaguer et al. [50]	EOP7	Corr, N	0.08	181	1	0.08	7.45E-02	8.02E-02	7.50E-02	Corr, N	Ego Orientation	Ego Orientation	Subjective	Athlete	Athlete	Elite	Elite
31	Balaguer et al. [50]	EOP8	Corr, N	0.03	181	1	0.03	7.49E-02	3.00E-02	7.50E-02	Corr, N	Ego Orientation	Ego Orientation	Subjective	Athlete	Team	Elite	Elite
32	Balaguer et al. [50]	EOP9	Corr, N	-0.01	181	1	-0.01	7.49E-02	-1.00E-02	7.50E-02	Corr, N	Ego Orientation	Ego Orientation	Subjective	Athlete	Team	Elite	Elite
33	Balaguer et al. [50]	TCP1	Corr, N	0.33	181	1	0.33	6.68E-02	0.342828	7.50E-02	Corr, N	Task Climate	Task Climate	Subjective	Athlete	Athlete	Elite	Elite
34	Balaguer et al. [50]	TCP10	Corr, N	0.37	181	1	0.37	6.47E-02	0.388423	7.50E-02	Corr, N	Task Climate	Task Climate	Subjective	Athlete	Team	Elite	Elite
35	Balaguer et al. [50]	TCP11	Corr, N	0.22	181	1	0.22	7.13E-02	0.223656	7.50E-02	Corr, N	Task Climate	Task Climate	Subjective	Athlete	Team	Elite	Elite
36	Balaguer et al. [50]	TCP12	Corr, N	0.12	181	1	0.12	7.39E-02	0.120581	7.50E-02	Corr, N	Task Climate	Task Climate	Subjective	Athlete	Team	Elite	Elite
37	Balaguer et al. [50]	TCP13	Corr, N	0.27	181	1	0.27	6.95E-02	0.276864	7.50E-02	Corr, N	Task Climate	Task Climate	Subjective	Athlete	Team	Elite	Elite

38	Balaguer et al. [50]	TCP14	Corr, N	0.15	181	1	0.15	7.33E-02	0.15114	7.50E-02	Corr, N	Task Climate	Task Climate	Subjective	Athlete	Team	Elite	Elite
39	Balaguer et al. [50]	TCP2	Corr, N	0.38	181	1	0.38	6.41E-02	0.40006	7.50E-02	Corr, N	Task Climate	Task Climate	Subjective	Athlete	Athlete	Elite	Elite
40	Balaguer et al. [50]	TCP3	Corr, N	0.43	181	1	0.43	6.11E-02	0.459897	7.50E-02	Corr, N	Task Climate	Task Climate	Subjective	Athlete	Athlete	Elite	Elite
41	Balaguer et al. [50]	TCP4	Corr, N	0.29	181	1	0.29	6.86E-02	0.298566	7.50E-02	Corr, N	Task Climate	Task Climate	Subjective	Athlete	Athlete	Elite	Elite
42	Balaguer et al. [50]	TCP5	Corr, N	0.3	181	1	0.3	6.82E-02	0.30952	7.50E-02	Corr, N	Task Climate	Task Climate	Subjective	Athlete	Athlete	Elite	Elite
43	Balaguer et al. [50]	TCP6	Corr, N	0.21	181	1	0.21	7.16E-02	0.213171	7.50E-02	Corr, N	Task Climate	Task Climate	Subjective	Athlete	Athlete	Elite	Elite
44	Balaguer et al. [50]	TCP7	Corr, N	0.09	181	1	0.09	7.43E-02	9.02E-02	7.50E-02	Corr, N	Task Climate	Task Climate	Subjective	Athlete	Athlete	Elite	Elite
45	Balaguer et al. [50]	TCP8	Corr, N	0.38	181	1	0.38	6.41E-02	0.40006	7.50E-02	Corr, N	Task Climate	Task Climate	Subjective	Athlete	Team	Elite	Elite
46	Balaguer et al. [50]	TCP9	Corr, N	0.44	181	1	0.44	6.04E-02	0.472231	7.50E-02	Corr, N	Task Climate	Task Climate	Subjective	Athlete	Team	Elite	Elite
47	Balaguer et al. [50]	TOP1	Corr, N	0.1	181	1	0.1	7.42E-02	0.100335	7.50E-02	Corr, N	Task Orientation	Task Orientation	Subjective	Athlete	Athlete	Elite	Elite
48	Balaguer et al. [50]	TOP10	Corr, N	0.11	181	1	0.11	7.40E-02	0.110447	7.50E-02	Corr, N	Task Orientation	Task Orientation	Subjective	Athlete	Team	Elite	Elite
49	Balaguer et al. [50]	TOP11	Corr, N	0.1	181	1	0.1	7.42E-02	0.100335	7.50E-02	Corr, N	Task Orientation	Task Orientation	Subjective	Athlete	Team	Elite	Elite
50	Balaguer et al. [50]	TOP12	Corr, N	0.09	181	1	0.09	7.43E-02	9.02E-02	7.50E-02	Corr, N	Task Orientation	Task Orientation	Subjective	Athlete	Team	Elite	Elite
51	Balaguer et al. [50]	TOP13	Corr, N	0.05	181	1	0.05	7.48E-02	5.00E-02	7.50E-02	Corr, N	Task Orientation	Task Orientation	Subjective	Athlete	Team	Elite	Elite

52	Balaguer et al. [50]	TOP14	Corr, N	0.07	181	1	0.07	7.46E-02	7.01E-02	7.50E-02	Corr, N	Task Orientation	Task Orientation	Subjective	Athlete	Team	Elite	Elite
53	Balaguer et al. [50]	TOP2	Corr, N	0.14	181	1	0.14	7.35E-02	0.140926	7.50E-02	Corr, N	Task Orientation	Task Orientation	Subjective	Athlete	Athlete	Elite	Elite
54	Balaguer et al. [50]	TOP3	Corr, N	0.21	181	1	0.21	7.16E-02	0.213171	7.50E-02	Corr, N	Task Orientation	Task Orientation	Subjective	Athlete	Athlete	Elite	Elite
55	Balaguer et al. [50]	TOP4	Corr, N	0.13	181	1	0.13	7.37E-02	0.13074	7.50E-02	Corr, N	Task Orientation	Task Orientation	Subjective	Athlete	Athlete	Elite	Elite
56	Balaguer et al. [50]	TOP5	Corr, N	0.06	181	1	0.06	7.47E-02	6.01E-02	7.50E-02	Corr, N	Task Orientation	Task Orientation	Subjective	Athlete	Athlete	Elite	Elite
57	Balaguer et al. [50]	TOP6	Corr, N	0.05	181	1	0.05	7.48E-02	5.00E-02	7.50E-02	Corr, N	Task Orientation	Task Orientation	Subjective	Athlete	Athlete	Elite	Elite
58	Balaguer et al. [50]	TOP7	Corr, N	0.03	181	1	0.03	7.49E-02	3.00E-02	7.50E-02	Corr, N	Task Orientation	Task Orientation	Subjective	Athlete	Athlete	Elite	Elite
59	Balaguer et al. [50]	TOP8	Corr, N	0.22	181	1	0.22	7.13E-02	0.223656	7.50E-02	Corr, N	Task Orientation	Task Orientation	Subjective	Athlete	Team	Elite	Elite
60	Balaguer et al. [50]	TOP9	Corr, N	0.21	181	1	0.21	7.16E-02	0.213171	7.50E-02	Corr, N	Task Orientation	Task Orientation	Subjective	Athlete	Team	Elite	Elite
61	Balaguer et al. [51]	ECP1	Corr, N	-0.01	219	1	-0.01	6.80E-02	-1.00E-02	6.80E-02	Corr, N	Ego Climate	Ego Climate	Subjective	Athlete	Athlete	Mix	Non-Elite
62	Balaguer et al. [51]	ECP2	Corr, N	-0.03	219	1	-0.03	6.80E-02	-3.00E-02	6.80E-02	Corr, N	Ego Climate	Ego Climate	Subjective	Athlete	Athlete	Mix	Non-Elite
63	Balaguer et al. [51]	ECP3	Corr, N	0.07	219	1	0.07	6.77E-02	7.01E-02	6.80E-02	Corr, N	Ego Climate	Ego Climate	Subjective	Athlete	Athlete	Mix	Non-Elite
64	Balaguer et al. [51]	ECP4	Corr, N	-0.05	219	1	-0.05	6.79E-02	-5.00E-02	6.80E-02	Corr, N	Ego Climate	Ego Climate	Subjective	Athlete	Athlete	Mix	Non-

																		Elite
65	Balaguer et al. [51]	ECP5	Corr, N	-0.16	219	1	-0.16	6.63E-02	-0.16139	6.80E-02	Corr, N	Ego Climate	Ego Climate	Subjective	Athlete	Athlete	Mix	Non-Elite
66	Balaguer et al. [51]	ECP6	Corr, N	0.13	219	1	0.13	0.066891	0.13074	6.80E-02	Corr, N	Ego Climate	Ego Climate	Subjective	Athlete	Athlete	Mix	Non-Elite
67	Balaguer et al. [51]	EOP1	Corr, N	-0.01	219	1	-0.01	6.80E-02	-1.00E-02	6.80E-02	Corr, N	Ego Orientation	Ego Orientation	Subjective	Athlete	Athlete	Mix	Non-Elite
68	Balaguer et al. [51]	EOP2	Corr, N	-0.01	219	1	-0.01	6.80E-02	-1.00E-02	6.80E-02	Corr, N	Ego Orientation	Ego Orientation	Subjective	Athlete	Athlete	Mix	Non-Elite
69	Balaguer et al. [51]	EOP3	Corr, N	0.08	219	1	0.08	6.76E-02	8.02E-02	6.80E-02	Corr, N	Ego Orientation	Ego Orientation	Subjective	Athlete	Athlete	Mix	Non-Elite
70	Balaguer et al. [51]	EOP4	Corr, N	0.06	219	1	0.06	6.78E-02	6.01E-02	6.80E-02	Corr, N	Ego Orientation	Ego Orientation	Subjective	Athlete	Athlete	Mix	Non-Elite
71	Balaguer et al. [51]	EOP5	Corr, N	0	219	1	0	6.80E-02	0	6.80E-02	Corr, N	Ego Orientation	Ego Orientation	Subjective	Athlete	Athlete	Mix	Non-Elite
72	Balaguer et al. [51]	EOP6	Corr, N	0.03	219	1	0.03	6.80E-02	3.00E-02	6.80E-02	Corr, N	Ego Orientation	Ego Orientation	Subjective	Athlete	Athlete	Mix	Non-Elite
73	Balaguer et al. [51]	TCP1	Corr, N	0.14	219	1	0.14	6.67E-02	0.140926	6.80E-02	Corr, N	Task Climate	Task Climate	Subjective	Athlete	Athlete	Mix	Non-Elite
74	Balaguer et al. [51]	TCP2	Corr, N	0.13	219	1	0.13	0.066891	0.13074	6.80E-02	Corr, N	Task Climate	Task Climate	Subjective	Athlete	Athlete	Mix	Non-Elite

75	Balaguer et al. [51]	TCP3	Corr, N	0.02	219	1	0.02	6.80E-02	2.00E-02	6.80E-02	Corr, N	Task Climate	Task Climate	Subjective	Athlete	Athlete	Mix	Non-Elite
76	Balaguer et al. [51]	TCP4	Corr, N	0.26	219	1	0.26	6.34E-02	0.266108	6.80E-02	Corr, N	Task Climate	Task Climate	Subjective	Athlete	Athlete	Mix	Non-Elite
77	Balaguer et al. [51]	TCP5	Corr, N	0.23	219	1	0.23	6.44E-02	0.234189	6.80E-02	Corr, N	Task Climate	Task Climate	Subjective	Athlete	Athlete	Mix	Non-Elite
78	Balaguer et al. [51]	TCP6	Corr, N	0.23	219	1	0.23	6.44E-02	0.234189	6.80E-02	Corr, N	Task Climate	Task Climate	Subjective	Athlete	Athlete	Mix	Non-Elite
79	Balaguer et al. [51]	TOP1	Corr, N	0.05	219	1	0.05	6.79E-02	5.00E-02	6.80E-02	Corr, N	Task Orientation	Task Orientation	Subjective	Athlete	Athlete	Mix	Non-Elite
80	Balaguer et al. [51]	TOP2	Corr, N	0.11	219	1	0.11	0.067218	0.110447	6.80E-02	Corr, N	Task Orientation	Task Orientation	Subjective	Athlete	Athlete	Mix	Non-Elite
81	Balaguer et al. [51]	TOP3	Corr, N	0.11	219	1	0.11	0.067218	0.110447	6.80E-02	Corr, N	Task Orientation	Task Orientation	Subjective	Athlete	Athlete	Mix	Non-Elite
82	Balaguer et al. [51]	TOP4	Corr, N	0.09	219	1	0.09	0.06749	9.02E-02	6.80E-02	Corr, N	Task Orientation	Task Orientation	Subjective	Athlete	Athlete	Mix	Non-Elite
83	Balaguer et al. [51]	TOP5	Corr, N	0.14	219	1	0.14	6.67E-02	0.140926	6.80E-02	Corr, N	Task Orientation	Task Orientation	Subjective	Athlete	Athlete	Mix	Non-Elite
84	Balaguer et al. [51]	TOP6	Corr, N	0.12	219	1	0.12	6.71E-02	0.120581	6.80E-02	Corr, N	Task Orientation	Task Orientation	Subjective	Athlete	Athlete	Mix	Non-Elite

85	Bono & Livi [52]	ECP1	Corr, N	-0.123	96	1	-0.123	0.102126	-0.12363	0.103695	Corr, N	Ego Climate	Ego Climate	Subjective	Athlete	Athlete	Mix	Non-Elite
86	Bono & Livi [52]	EOP1	Corr, N	-0.131	96	1	-0.131	0.101916	-0.13176	0.103695	Corr, N	Ego Orientation	Ego Orientation	Subjective	Athlete	Athlete	Mix	Non-Elite
87	Bono & Livi [52]	TCP1	Corr, N	0.283	96	1	0.283	0.09539	0.29094	0.103695	Corr, N	Task Climate	Task Climate	Subjective	Athlete	Athlete	Mix	Non-Elite
88	Bono & Livi [52]	TOP1	Corr, N	0.265	96	1	0.265	9.64E-02	0.271478	0.103695	Corr, N	Task Orientation	Task Orientation	Subjective	Athlete	Athlete	Mix	Non-Elite
89	Bortoli et al. [53]	ECP1	Corr, N	-0.026	320	1	-0.026	0.056128	-2.60E-02	0.056166	Corr, N	Ego Climate	Ego Climate	Subjective	Coach	Athlete	Youth	Non-Elite
90	Bortoli et al. [53]	EOP1	Corr, N	0.024	320	1	0.024	5.61E-02	2.40E-02	0.056166	Corr, N	Ego Orientation	Ego Orientation	Subjective	Coach	Athlete	Youth	Non-Elite
91	Bortoli et al. [53]	TCP1	Corr, N	0.076	320	1	0.076	5.58E-02	7.61E-02	0.056166	Corr, N	Task Climate	Task Climate	Subjective	Coach	Athlete	Youth	Non-Elite
92	Bortoli et al. [53]	TOP1	Corr, N	0.178	320	1	0.178	5.44E-02	0.179916	0.056166	Corr, N	Task Orientation	Task Orientation	Subjective	Coach	Athlete	Youth	Non-Elite
93	Boyd & Callaghan [54]	EOP1	Corr, N	0.09	91	1	0.09	0.105737	9.02E-02	0.1066	Corr, N	Ego Orientation	Ego Orientation	Subjective	Athlete	Athlete	Youth	Non-Elite
94	Boyd & Callaghan [54]	TOP1	Corr, N	0	91	1	0	0.1066	0	0.1066	Corr, N	Task Orientation	Task Orientation	Subjective	Athlete	Athlete	Youth	Non-Elite

95	Branco et al. [55]	EOP1	Corr, N	0.18	291	1	0.18	5.70E-02	0.181983	0.058926	Corr, N	Ego Orientation	Ego Orientation	Subjective	Athlete	Athlete	Youth/Intermediate	Non-Elite
96	Branco et al. [55]	EOP2	Corr, N	0.17	291	1	0.17	5.72E-02	0.171667	0.058926	Corr, N	Ego Orientation	Ego Orientation	Subjective	Athlete	Team	Youth/Intermediate	Non-Elite
97	Branco et al. [55]	TOP1	Corr, N	0.25	291	1	0.25	0.055243	0.255413	0.058926	Corr, N	Task Orientation	Task Orientation	Subjective	Athlete	Athlete	Youth/Intermediate	Non-Elite
98	Branco et al. [55]	TOP2	Corr, N	0.13	291	1	0.13	5.79E-02	0.13074	0.058926	Corr, N	Task Orientation	Task Orientation	Subjective	Athlete	Team	Youth/Intermediate	Non-Elite
99	Cervello et al. [56]	ECP1	Corr, N	-0.05	151	1	-0.05	8.20E-02	-5.00E-02	8.22E-02	Corr, N	Ego Climate	Ego Climate	Subjective	Coach	Athlete	Intermediate/Advanced	Non-Elite
100	Cervello et al. [56]	ECP2	Corr, N	0.09	151	1	0.09	0.081534	9.02E-02	8.22E-02	Corr, N	Ego Climate	Ego Climate	Subjective	Athlete	Athlete	Intermediate/Advanced	Non-Elite
101	Cervello et al. [56]	ECP3	Corr, N	0.36	151	1	0.36	7.15E-02	0.376886	8.22E-02	Corr, N	Ego Climate	Ego Climate	Subjective	Coach	Athlete	Intermediate/Advanced	Non-Elite
102	Cervello et al. [56]	ECP4	Corr, N	0.05	151	1	0.05	8.20E-02	5.00E-02	8.22E-02	Corr, N	Ego Climate	Ego Climate	Subjective	Athlete	Athlete	Intermediate/Advanced	Non-Elite
103	Cervello et al. [56]	EOP1	Corr, N	0.16	151	1	0.16	8.01E-02	0.161387	8.22E-02	Corr, N	Ego Orientation	Ego Orientation	Subjective	Coach	Athlete	Intermediate/Advanced	Non-Elite
104	Cervello et al. [56]	EOP2	Corr, N	0.1	151	1	0.1	8.14E-02	0.100335	8.22E-02	Corr, N	Ego Orientation	Ego Orientation	Subjective	Athlete	Athlete	Intermediate/Advanced	Non-Elite

105	Cervello et al. [56]	TCP1	Corr, N	0.05	151	1	0.05	8.20E-02	5.00E-02	8.22E-02	Corr, N	Task Climate	Task Climate	Subjective	Coach	Athlete	Intermediate/ Advanced	Non-Elite
106	Cervello et al. [56]	TCP2	Corr, N	0.18	151	1	0.18	7.95E-02	0.181983	8.22E-02	Corr, N	Task Climate	Task Climate	Subjective	Athlete	Athlete	Intermediate/ Advanced	Non-Elite
107	Cervello et al. [56]	TCP3	Corr, N	0.55	151	1	0.55	5.73E-02	0.618381	8.22E-02	Corr, N	Task Climate	Task Climate	Subjective	Coach	Athlete	Intermediate/ Advanced	Non-Elite
108	Cervello et al. [56]	TCP4	Corr, N	0.31	151	1	0.31	7.43E-02	0.320545	8.22E-02	Corr, N	Task Climate	Task Climate	Subjective	Athlete	Athlete	Intermediate/ Advanced	Non-Elite
109	Cervello et al. [56]	TOP1	Corr, N	0.03	151	1	0.03	8.21E-02	3.00E-02	8.22E-02	Corr, N	Task Orientation	Task Orientation	Subjective	Coach	Athlete	Intermediate/ Advanced	Non-Elite
110	Cervello et al. [56]	TOP2	Corr, N	0.25	151	1	0.25	7.71E-02	0.255413	8.22E-02	Corr, N	Task Orientation	Task Orientation	Subjective	Athlete	Athlete	Intermediate/ Advanced	Non-Elite
111	Cumming et al. [57]	ECP1	Corr, N	0.02	268	1	0.02	6.14E-02	2.00E-02	6.14E-02	Corr, N	Ego Climate	Ego Climate	Objective	Historical	Team	Youth	Non-Elite
112	Cumming et al. [57]	TCP1	Corr, N	0.14	268	1	0.14	6.02E-02	0.140926	6.14E-02	Corr, N	Task Climate	Task Climate	Objective	Historical	Team	Youth	Non-Elite
113	Dewar & Kavussanu [58]	EOP1	Corr, N	0.19	358	1	0.19	5.12E-02	0.192337	5.31E-02	Corr, N	Ego Orientation	Ego Orientation	Subjective	Athlete	Athlete	Mix	Non-Elite
114	Dewar & Kavussanu [58]	EOP2	Corr, N	0.04	358	1	0.04	0.05299	4.00E-02	5.31E-02	Corr, N	Ego Orientation	Ego Orientation	Objective	Historical	Team	Mix	Non-Elite

115	Dewar & Kavussanu [58]	TOP1	Corr, N	0.26	358	1	0.26	4.95E-02	0.266108	5.31E-02	Corr, N	Task Orientation	Task Orientation	Subjective	Athlete	Athlete	Mix	Non-Elite
116	Dewar & Kavussanu [58]	TOP2	Corr, N	0.06	358	1	0.06	5.29E-02	6.01E-02	5.31E-02	Corr, N	Task Orientation	Task Orientation	Objective	Historical	Team	Mix	Non-Elite
117	e Silva et al. [59]	EOP1	Independent groups (means, SD's)				0.340123	7.31E-02	0.354232	8.26E-02	Selection	Ego Orientation	Ego Orientation	Subjective	Coach	Athlete	Youth	Non-Elite
118	e Silva et al. [59]	TOP1	Independent groups (means, SD's)				8.75E-02	9.28E-02	8.77E-02	9.35E-02	Selection	Task Orientation	Task Orientation	Subjective	Coach	Athlete	Youth	Non-Elite
119	Elferink-Gemser et al. [60]	EOP1	Corr, N	0.467	63	1	0.467	0.100944	0.506227	0.129099	Corr, N	Ego Orientation	Ego Orientation	Objective	Historical	Athlete	Advanced	Non-Elite
120	Elferink-Gemser et al. [60]	TOP1	Corr, N	0.135	63	1	0.135	0.126747	0.135829	0.129099	Corr, N	Task Orientation	Task Orientation	Objective	Historical	Athlete	Advanced	Non-Elite
121	Farkhondeh & Moghaddam [61]	TOP1	Corr, N	0.06	150	1	0.06	0.082182	6.01E-02	8.25E-02	Corr, N	Task Orientation	Task Orientation	Objective	Historical	Athlete	Youth	Non-Elite
122	Figueiredo et al. [62] s1	EOP1	Independent groups (means, SD's)				-0.11802	0.120954	-0.11857	0.122662	Selection	Ego Orientation	Ego Orientation	Subjective	Coach	Athlete	Youth	Non-Elite
123	Figueiredo et al. [62] s1	TOP1	Independent groups (means, SD's)				0	0.123091	0	0.123091	Selection	Task Orientation	Task Orientation	Subjective	Coach	Athlete	Youth	Non-Elite
124	Figueiredo et al. [62] s2	EOP1	Independent groups (means, SD's)				0	0.11547	0	0.11547	Selection	Ego Orientation	Ego Orientation	Subjective	Coach	Athlete	Youth	Non-

																		Elite
125	Figueiredo et al. [62] s2	TOP1	Independent groups (means, SD's)				0	0.132453	0	0.132453	Selection	Task Orientation	Task Orientation	Subjective	Coach	Athlete	Youth	Non-Elite
126	García-Calvo et al. [63]	ECP1	Corr, N	-0.2	377	1	-0.2	4.96E-02	-0.20273	5.17E-02	Corr, N	Ego Climate	Ego Climate	Subjective	Athlete	Athlete	Advanced	Non-Elite
127	García-Calvo et al. [63]	ECP10	Corr, N	0.04	339	1	0.04	5.45E-02	4.00E-02	5.46E-02	Corr, N	Ego Climate	Ego Climate	Subjective	Athlete	Athlete	Advanced	Non-Elite
128	García-Calvo et al. [63]	ECP11	Corr, N	0	303	1	0	5.77E-02	0	5.77E-02	Corr, N	Ego Climate	Ego Climate	Subjective	Athlete	Athlete	Advanced	Non-Elite
129	García-Calvo et al. [63]	ECP12	Corr, N	-0.09	303	1	-0.09	0.057267	-9.02E-02	5.77E-02	Corr, N	Ego Climate	Ego Climate	Subjective	Athlete	Athlete	Advanced	Non-Elite
130	García-Calvo et al. [63]	ECP2	Corr, N	-0.17	339	1	-0.17	5.30E-02	-0.17167	5.46E-02	Corr, N	Ego Climate	Ego Climate	Subjective	Athlete	Athlete	Advanced	Non-Elite
131	García-Calvo et al. [63]	ECP3	Corr, N	-0.3	303	1	-0.3	5.25E-02	-0.30952	5.77E-02	Corr, N	Ego Climate	Ego Climate	Subjective	Athlete	Athlete	Advanced	Non-Elite
132	García-Calvo et al. [63]	ECP4	Corr, N	-0.06	303	1	-0.06	5.75E-02	-6.01E-02	5.77E-02	Corr, N	Ego Climate	Ego Climate	Subjective	Athlete	Athlete	Advanced	Non-Elite
133	García-Calvo et al. [63]	ECP5	Corr, N	-0.06	303	1	-0.06	5.75E-02	-6.01E-02	5.77E-02	Corr, N	Ego Climate	Ego Climate	Subjective	Athlete	Athlete	Advanced	Non-Elite
134	García-Calvo et al. [63]	ECP6	Corr, N	-0.07	303	1	-0.07	5.75E-02	-7.01E-02	5.77E-02	Corr, N	Ego Climate	Ego Climate	Subjective	Athlete	Athlete	Advanced	Non-Elite

135	García-Calvo et al. [63]	ECP7	Corr, N	-0.12	339	1	-0.12	5.38E-02	-0.12058	5.46E-02	Corr, N	Ego Climate	Ego Climate	Subjective	Athlete	Athlete	Advanced	Non-Elite
136	García-Calvo et al. [63]	ECP8	Corr, N	-0.15	303	1	-0.15	5.64E-02	-0.15114	5.77E-02	Corr, N	Ego Climate	Ego Climate	Subjective	Athlete	Athlete	Advanced	Non-Elite
137	García-Calvo et al. [63]	ECP9	Corr, N	0.04	303	1	0.04	5.76E-02	4.00E-02	5.77E-02	Corr, N	Ego Climate	Ego Climate	Subjective	Athlete	Athlete	Advanced	Non-Elite
138	García-Calvo et al. [63]	TCP1	Corr, N	0.19	377	1	0.19	4.98E-02	0.192337	5.17E-02	Corr, N	Task Climate	Task Climate	Subjective	Athlete	Athlete	Advanced	Non-Elite
139	García-Calvo et al. [63]	TCP10	Corr, N	0.1	339	1	0.1	5.40E-02	0.100335	5.46E-02	Corr, N	Task Climate	Task Climate	Subjective	Athlete	Athlete	Advanced	Non-Elite
140	García-Calvo et al. [63]	TCP11	Corr, N	0.09	303	1	0.09	0.057267	9.02E-02	5.77E-02	Corr, N	Task Climate	Task Climate	Subjective	Athlete	Athlete	Advanced	Non-Elite
141	García-Calvo et al. [63]	TCP12	Corr, N	0.07	303	1	0.07	5.75E-02	7.01E-02	5.77E-02	Corr, N	Task Climate	Task Climate	Subjective	Athlete	Athlete	Advanced	Non-Elite
142	García-Calvo et al. [63]	TCP2	Corr, N	0.22	339	1	0.22	5.19E-02	0.223656	5.46E-02	Corr, N	Task Climate	Task Climate	Subjective	Athlete	Athlete	Advanced	Non-Elite
143	García-Calvo et al. [63]	TCP3	Corr, N	0.28	303	1	0.28	5.32E-02	0.287682	5.77E-02	Corr, N	Task Climate	Task Climate	Subjective	Athlete	Athlete	Advanced	Non-Elite
144	García-Calvo et al. [63]	TCP4	Corr, N	0.05	303	1	0.05	5.76E-02	5.00E-02	5.77E-02	Corr, N	Task Climate	Task Climate	Subjective	Athlete	Athlete	Advanced	Non-Elite

145	García-Calvo et al. [63]	TCP5	Corr, N	0	303	1	0	5.77E-02	0	5.77E-02	Corr, N	Task Climate	Task Climate	Subjective	Athlete	Athlete	Advanced	Non-Elite
146	García-Calvo et al. [63]	TCP6	Corr, N	0.04	303	1	0.04	5.76E-02	4.00E-02	5.77E-02	Corr, N	Task Climate	Task Climate	Subjective	Athlete	Athlete	Advanced	Non-Elite
147	García-Calvo et al. [63]	TCP7	Corr, N	0.12	339	1	0.12	5.38E-02	0.120581	5.46E-02	Corr, N	Task Climate	Task Climate	Subjective	Athlete	Athlete	Advanced	Non-Elite
148	García-Calvo et al. [63]	TCP8	Corr, N	0.09	303	1	0.09	0.057267	9.02E-02	5.77E-02	Corr, N	Task Climate	Task Climate	Subjective	Athlete	Athlete	Advanced	Non-Elite
149	García-Calvo et al. [63]	TCP9	Corr, N	0.26	303	1	0.26	5.38E-02	0.266108	5.77E-02	Corr, N	Task Climate	Task Climate	Subjective	Athlete	Athlete	Advanced	Non-Elite
150	Holgado et al. [64]	ECP1	Corr, N	0.203	511	1	0.203	4.25E-02	0.20586	4.44E-02	Corr, N	Ego Climate	Ego Climate	Subjective	Athlete	Athlete	Elite	Elite
151	Holgado et al. [64]	ECP2	Corr, N	-0.071	511	1	-0.071	4.41E-02	-7.11E-02	4.44E-02	Corr, N	Ego Climate	Ego Climate	Subjective	Athlete	Athlete	Elite	Elite
152	Holgado et al. [64]	TCP1	Corr, N	0.185	511	1	0.185	4.28E-02	0.187155	4.44E-02	Corr, N	Task Climate	Task Climate	Subjective	Athlete	Athlete	Elite	Elite
153	Holgado et al. [64]	TCP2	Corr, N	0.413	511	1	0.413	3.68E-02	0.439223	4.44E-02	Corr, N	Task Climate	Task Climate	Subjective	Athlete	Athlete	Elite	Elite
154	Höner & Feichtinger 2016 ob1a	EOP6	Corr, N	0	2677	1	0	1.93E-02	0	1.93E-02	Corr, N	Ego Orientation	Ego Orientation	Objective	Historical	Athlete	Advanced	Non-Elite
155	Höner & Feichtinger [65] 1a	TOP6	Corr, N	-0.02	2677	1	-0.02	1.93E-02	-2.00E-02	1.93E-02	Corr, N	Task Orientation	Task Orientation	Objective	Historical	Athlete	Advanced	Non-Elite

156	Höner & Feichtinger [65] ob1b	EOP3	Independent groups (means, SD's)			-2.54E-02	3.32E-02	-2.54E-02	0.033236	Selection	Ego Orientation	Ego Orientation	Subjective	Coach	Athlete	Advanced	Non-Elite
157	Höner & Feichtinger [65] ob1b	EOP4	Independent groups (means, SD's)			-2.72E-02	5.83E-02	-2.72E-02	5.83E-02	Selection	Ego Orientation	Ego Orientation	Subjective	Coach	Athlete	Advanced	Non-Elite
158	Höner & Feichtinger [65] ob1b	EOP5	Independent groups (means, SD's)			0	0.035921	0	0.035921	Selection	Ego Orientation	Ego Orientation	Subjective	Coach	Athlete	Advanced	Non-Elite
159	Höner & Feichtinger [65] ob1b	TOP3	Independent groups (means, SD's)			2.44E-02	3.32E-02	0.024432	3.32E-02	Selection	Task Orientation	Task Orientation	Subjective	Coach	Athlete	Advanced	Non-Elite
160	Höner & Feichtinger [65] ob1b	TOP4	Independent groups (means, SD's)			3.55E-02	5.82E-02	3.55E-02	5.83E-02	Selection	Task Orientation	Task Orientation	Subjective	Coach	Athlete	Advanced	Non-Elite
161	Höner & Feichtinger [65] ob1b	TOP5	Independent groups (means, SD's)			5.98E-03	3.59E-02	0.005978	3.59E-02	Selection	Task Orientation	Task Orientation	Subjective	Coach	Athlete	Advanced	Non-Elite
162	Höner & Feichtinger [65] ob2	EOP1	Odds ratio			2.37E-02	2.24E-02	2.38E-02	2.25E-02	Selection	Ego Orientation	Ego Orientation	Objective	Coach	Athlete	Advanced	Non-Elite
163	Höner & Feichtinger [65] ob2	TOP2	Odds ratio			6.14E-02	2.40E-02	6.15E-02	2.41E-02	Selection	Task Orientation	Task Orientation	Objective	Historical	Athlete	Advanced	Non-Elite

164	Huijgen et al. [66]	EOP1	Independent groups (means, SD's)				0.023462	9.40E-02	2.35E-02	9.41E-02	Selection	Ego Orientation	Ego Orientation	Subjective	Coach	Athlete	Advanced	Non-Elite
165	Huijgen et al. [66]	TOP1	Independent groups (means, SD's)				9.53E-02	9.30E-02	9.55E-02	9.39E-02	Selection	Task Orientation	Task Orientation	Subjective	Coach	Athlete	Advanced	Non-Elite
166	Jeong [67]	EOP1	Corr, N	0.602	303	1	0.602	3.68E-02	0.696278	5.77E-02	Corr, N	Ego Orientation	Ego Orientation	Subjective	Athlete	Athlete	Elite	Elite
167	Jeong [67]	TOP1	Corr, N	0.575	303	1	0.575	3.86E-02	0.654961	5.77E-02	Corr, N	Task Orientation	Task Orientation	Subjective	Athlete	Athlete	Elite	Elite
168	Kim [68]	EOP1	Independent groups (means, SD's)				0.113757	0.102575	0.114252	0.103919	Selection	Ego Orientation	Ego Orientation	Objective	Historical	Athlete	Intermediate/advanced	Non-Elite
169	Kim [68]	TOP1	Independent groups (means, SD's)				0.182443	9.99E-02	0.184508	0.103386	Selection	Task Orientation	Task Orientation	Objective	Historical	Athlete	Intermediate/advanced	Non-Elite
170	Knoblochova et al. [69]	EOP1	Corr, N	0.228	128	1	0.228	8.48E-02	0.232079	8.94E-02	Corr, N	Ego Orientation	Ego Orientation	Objective	Historical	Athlete	Elite	Elite
171	Knoblochova et al. [69]	TOP1	Corr, N	0.065	128	1	0.065	8.91E-02	6.51E-02	8.94E-02	Corr, N	Task Orientation	Task Orientation	Objective	Historical	Athlete	Elite	Elite
172	Lemyre et al. [70]	ECP1	Corr, N	-0.07	141	1	-0.07	8.47E-02	-7.01E-02	8.51E-02	Corr, N	Ego Climate	Ego Climate	Subjective	Athlete	Athlete	Elite	Elite
173	Lemyre et al. [70]	ECP2	Corr, N	-0.19	141	1	-0.19	8.21E-02	-0.19234	8.51E-02	Corr, N	Ego Climate	Ego Climate	Subjective	Athlete	Athlete	Elite	Elite
174	Lemyre et al. [70]	EOP1	Corr, N	0.05	141	1	0.05	8.49E-02	5.00E-02	8.51E-02	Corr, N	Ego Orientation	Ego Orientation	Subjective	Athlete	Athlete	Elite	Elite
175	Lemyre et al. [70]	EOP2	Corr, N	-0.01	141	1	-0.01	8.51E-02	-1.00E-02	8.51E-02	Corr, N	Ego Orientation	Ego Orientation	Subjective	Athlete	Athlete	Elite	Elite

176	Lemyre et al. [70]	TCP1	Corr, N	0.17	141	1	0.17	8.27E-02	0.171667	8.51E-02	Corr, N	Task Climate	Task Climate	Subjective	Athlete	Athlete	Elite	Elite
177	Lemyre et al. [70]	TCP2	Corr, N	0.22	141	1	0.22	8.10E-02	0.223656	8.51E-02	Corr, N	Task Climate	Task Climate	Subjective	Athlete	Athlete	Elite	Elite
178	Lemyre et al. [70]	TOP1	Corr, N	0.17	141	1	0.17	8.27E-02	0.171667	8.51E-02	Corr, N	Task Orientation	Task Orientation	Subjective	Athlete	Athlete	Elite	Elite
179	Lemyre et al. [70]	TOP2	Corr, N	0.19	141	1	0.19	8.21E-02	0.192337	8.51E-02	Corr, N	Task Orientation	Task Orientation	Subjective	Athlete	Athlete	Elite	Elite
180	Peng & Zhang [71] S1	EOP1	Corr, N	-0.18	81	1	-0.18	0.109559	-0.18198	0.113228	Corr, N	Ego Orientation	Ego Orientation	Objective	Historical	Athlete	Advanced	Non-Elite
181	Peng & Zhang [71] S1	TOP1	Corr, N	0.05	81	1	0.05	0.112945	5.00E-02	0.113228	Corr, N	Task Orientation	Task Orientation	Objective	Historical	Athlete	Advanced	Non-Elite
182	Peng & Zhang [71] S2	EOP1	Corr, N	-0.19	81	1	-0.19	0.10914	-0.19234	0.113228	Corr, N	Ego Orientation	Ego Orientation	Objective	Historical	Athlete	Advanced	Non-Elite
183	Peng & Zhang [71] S2	TOP1	Corr, N	0.16	81	1	0.16	0.110329	0.161387	0.113228	Corr, N	Task Orientation	Task Orientation	Objective	Historical	Athlete	Advanced	Non-Elite
184	Santos-Rosa et al. [72]	ECP1	Corr, N	0.1	258	1	0.1	6.20E-02	0.100335	0.062622	Corr, N	Ego Climate	Ego Climate	Subjective	Athlete/ Coach	Athlete	Advanced	Non-Elite
185	Santos-Rosa et al. [72]	TCP1	Corr, N	-0.01	258	1	-0.01	6.26E-02	-1.00E-02	0.062622	Corr, N	Task Climate	Task Climate	Subjective	Athlete/ Coach	Athlete	Advanced	Non-Elite
186	Smith et al. [73]	ECP1	Corr, N	0	223	1	0	6.74E-02	0	6.74E-02	Corr, N	Ego Climate	Ego Climate	Subjective	Athlete	Athlete	Youth	Non-Elite

187	Smith et al. [73]	EOP1	Corr, N	-0.05	223	1	-0.05	6.73E-02	-5.00E-02	6.74E-02	Corr, N	Ego Orientation	Ego Orientation	Subjective	Athlete	Athlete	Youth	Non-Elite
188	Smith et al. [73]	TCP1	Corr, N	0.23	223	1	0.23	6.39E-02	0.234189	6.74E-02	Corr, N	Task Climate	Task Climate	Subjective	Athlete	Athlete	Youth	Non-Elite
189	Smith et al. [73]	TOP1	Corr, N	0.28	223	1	0.28	6.21E-02	0.287682	6.74E-02	Corr, N	Task Orientation	Task Orientation	Subjective	Athlete	Athlete	Youth	Non-Elite
190	Tello et al. [74]	ECP1	Corr, N	0.203	511	1	0.203	4.25E-02	0.20586	4.44E-02	Corr, N	Ego Climate	Ego Climate	Subjective	Athlete	Athlete	Mix	Non-Elite
191	Tello et al. [74]	ECP2	Corr, N	-0.071	511	1	-0.071	4.41E-02	-7.11E-02	4.44E-02	Corr, N	Ego Climate	Ego Climate	Subjective	Athlete	Athlete	Mix	Non-Elite
192	Tello et al. [74]	EOP1	Corr, N	0.29	511	1	0.29	4.06E-02	0.298566	4.44E-02	Corr, N	Ego Orientation	Ego Orientation	Subjective	Athlete	Athlete	Mix	Non-Elite
193	Tello et al. [74]	EOP2	Corr, N	-0.009	511	1	-0.009	4.44E-02	-9.00E-03	4.44E-02	Corr, N	Ego Orientation	Ego Orientation	Subjective	Athlete	Athlete	Mix	Non-Elite
194	Tello et al. [74]	TCP1	Corr, N	0.185	511	1	0.185	4.28E-02	0.187155	4.44E-02	Corr, N	Task Climate	Task Climate	Subjective	Athlete	Athlete	Mix	Non-Elite
195	Tello et al. [74]	TCP2	Corr, N	0.413	511	1	0.413	3.68E-02	0.439223	4.44E-02	Corr, N	Task Climate	Task Climate	Subjective	Athlete	Athlete	Mix	Non-Elite
196	Tello et al. [74]	TOP1	Corr, N	0.156	511	1	0.156	4.33E-02	0.157284	4.44E-02	Corr, N	Task Orientation	Task Orientation	Subjective	Athlete	Athlete	Mix	Non-Elite

197	Tello et al. [74]	TOP2	Corr, N	0.502	511	1	0.502	3.32E-02	0.551976	4.44E-02	Corr, N	Task Orientation	Task Orientation	Subjective	Athlete	Athlete	Mix	Non-Elite
198	Tenenbaum et al. [75]	EOP1	Corr, N	0.374	28	1	0.374	0.172025	0.393066	0.2	Corr, N	Ego Orientation	Ego Orientation	Objective	Historical	Athlete	Youth/Intermediate	Non-Elite
199	Tenenbaum et al. [75]	TOP1	Corr, N	0.4	28	1	0.4	0.168	0.423649	0.2	Corr, N	Task Orientation	Task Orientation	Objective	Historical	Athlete	Youth/Intermediate	Non-Elite
200	van de Pol & Kavussanu [76]	EOP1	Corr, N	0.1	116	1	0.1	9.31E-02	0.100335	9.41E-02	Corr, N	Ego Orientation	Ego Orientation	Subjective	Athlete	Athlete	Mix	Non-Elite
201	van de Pol & Kavussanu [76]	EOP2	Corr, N	0.25	116	1	0.25	8.82E-02	0.255413	9.41E-02	Corr, N	Ego Orientation	Ego Orientation	Objective	Historical	Athlete	Mix	Non-Elite
202	van de Pol & Kavussanu [76]	TOP1	Corr, N	0.21	116	1	0.21	8.99E-02	0.213171	9.41E-02	Corr, N	Task Orientation	Task Orientation	Subjective	Athlete	Athlete	Mix	Non-Elite
203	van de Pol & Kavussanu [76]	TOP2	Corr, N	0.24	116	1	0.24	8.87E-02	0.244774	9.41E-02	Corr, N	Task Orientation	Task Orientation	Objective	Historical	Athlete	Mix	Non-Elite
204	Van-Yperen & Duda [76]	EOP1	Corr, N	-0.02	75	1	-0.02	0.117804	-2.00E-02	0.117851	Corr, N	Ego Orientation	Ego Orientation	Subjective	Athlete	Athlete	Advanced	Non-Elite
205	Van-Yperen & Duda [76]	EOP2	Corr, N	0.01	75	1	0.01	0.117839	1.00E-02	0.117851	Corr, N	Ego Orientation	Ego Orientation	Subjective	Coach	Athlete	Advanced	Non-Elite
206	Van-Yperen & Duda [76]	EOP3	Corr, N	0.07	69	1	0.07	0.122488	7.01E-02	0.123091	Corr, N	Ego Orientation	Ego Orientation	Subjective	Athlete	Athlete	Advanced	Non-Elite

207	Van-Yperen & Duda [76]	EOP4	Corr, N	0.04	69	1	0.04	0.122895	4.00E-02	0.123091	Corr, N	Ego Orientation	Ego Orientation	Subjective	Coach	Athlete	Advanced	Non-Elite
208	Van-Yperen & Duda [76]	TOP1	Corr, N	0.31	75	1	0.31	0.106526	0.320545	0.117851	Corr, N	Task Orientation	Task Orientation	Subjective	Athlete	Athlete	Advanced	Non-Elite
209	Van-Yperen & Duda [76]	TOP2	Corr, N	0.15	75	1	0.15	0.115199	0.15114	0.117851	Corr, N	Task Orientation	Task Orientation	Subjective	Coach	Athlete	Advanced	Non-Elite
210	Van-Yperen & Duda [76]	TOP3	Corr, N	0.12	69	1	0.12	0.121319	0.120581	0.123091	Corr, N	Task Orientation	Task Orientation	Subjective	Athlete	Athlete	Advanced	Non-Elite
211	Van-Yperen & Duda [76]	TOP4	Corr, N	0.08	69	1	0.08	0.122304	8.02E-02	0.123091	Corr, N	Task Orientation	Task Orientation	Subjective	Coach	Athlete	Advanced	Non-Elite
212	Vazou [78]	ECP1	Corr, N	-0.11	483	1	-0.11	4.51E-02	-0.11045	4.56E-02	Corr, N	Ego Climate	Ego Climate	Subjective	Coach	Athlete	Youth/Intermediate	Non-Elite
213	Vazou [78]	ECP2	Corr, N	-0.06	483	1	-0.06	4.55E-02	-6.01E-02	4.56E-02	Corr, N	Ego Climate	Ego Climate	Subjective	Coach	Athlete	Youth/Intermediate	Non-Elite
214	Vazou [78]	EOP1	Corr, N	-0.06	483	1	-0.06	4.55E-02	-6.01E-02	4.56E-02	Corr, N	Ego Orientation	Ego Orientation	Subjective	Coach	Athlete	Youth/Intermediate	Non-Elite
215	Vazou [78]	TCP1	Corr, N	0.1	483	1	0.1	4.52E-02	0.100335	4.56E-02	Corr, N	Task Climate	Task Climate	Subjective	Coach	Athlete	Youth/Intermediate	Non-Elite
216	Vazou [78]	TCP2	Corr, N	0.17	483	1	0.17	4.43E-02	0.171667	4.56E-02	Corr, N	Task Climate	Task Climate	Subjective	Coach	Athlete	Youth/Intermediate	Non-Elite

217	Vazou [78]	TOP1	Corr, N	0.12	483	1	0.12	4.50E-02	0.120581	4.56E-02	Corr, N	Task Orientation	Task Orientation	Subjective	Coach	Athlete	Youth/Intermediate	Non-Elite

Supplemental Figures S1–S6. Funnel plots.

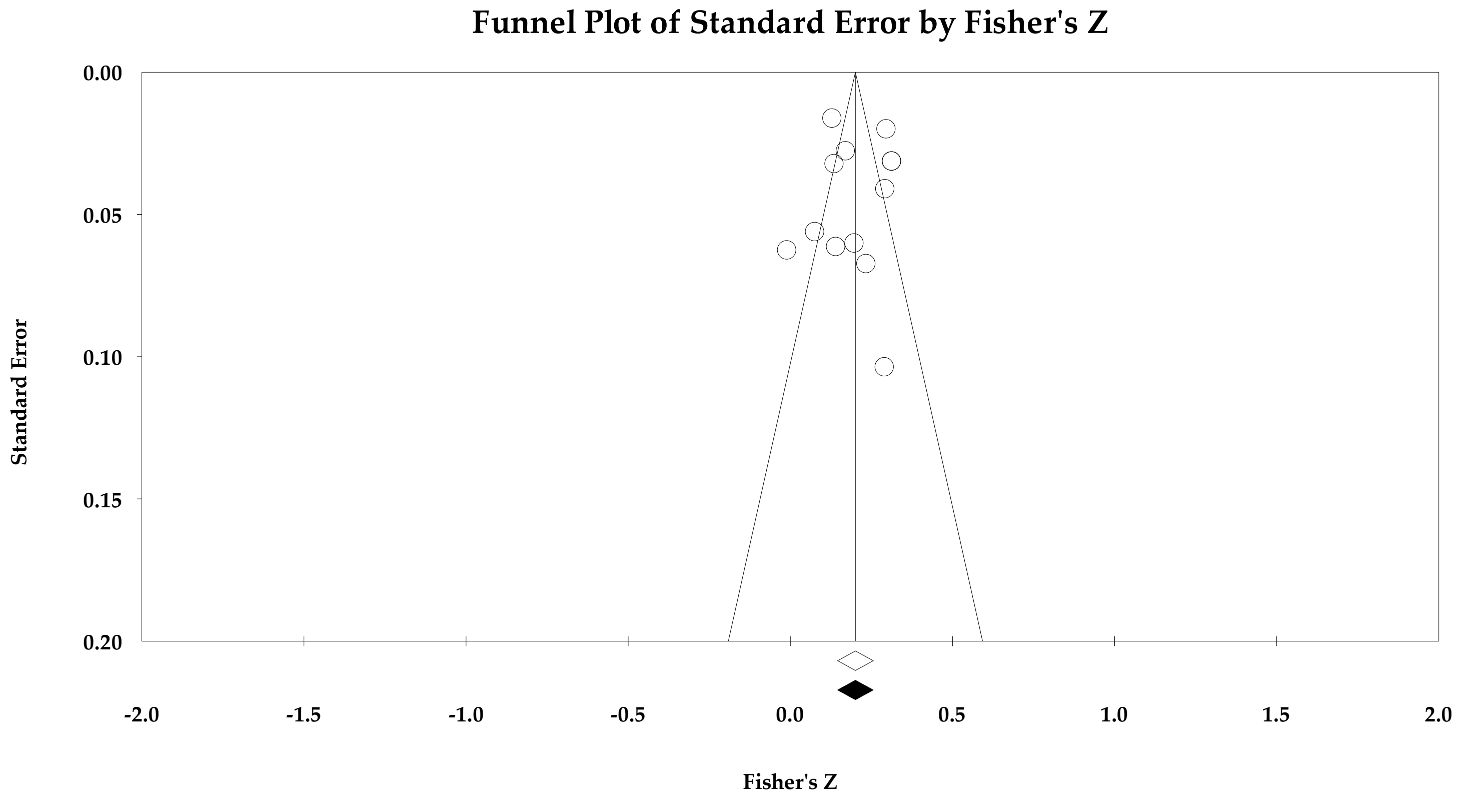

Supplemental Figure S1. Task climate and performance random effects plots trimmed and filled. The open circles are the data points, and the filled circles are the result of the trim and fill analysis. The clear rhombus is the mean effect size, and the filled rhombus is the trim and filled mean effect size.

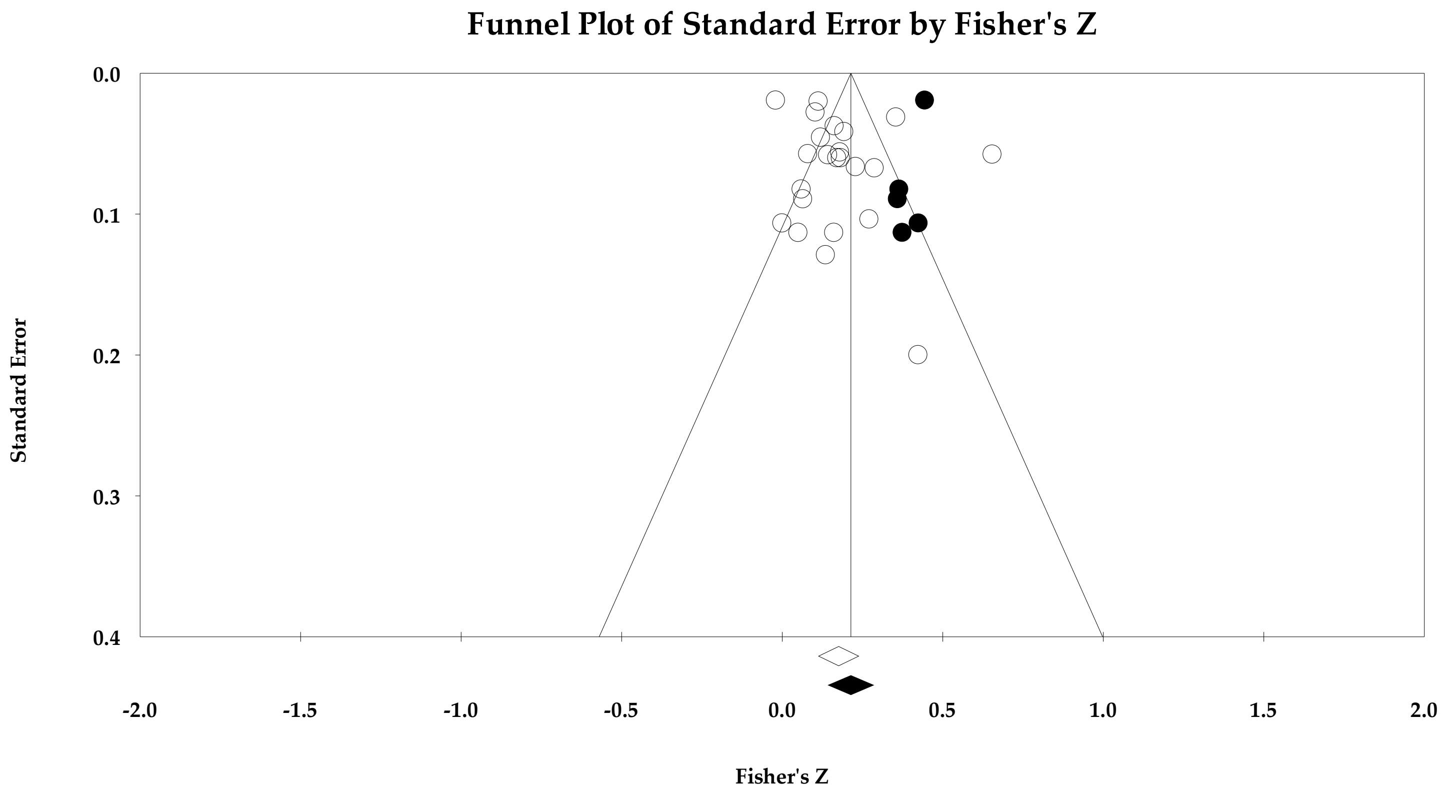

Supplemental Figure S2. Task orientation and performance random effects plots trimmed and filled (*r* samples). The open circles are the data points, and the filled circles are the result of the trim and fill analysis. The clear rhombus is the mean effect size, and the filled rhombus is the trim and filled mean effect size.

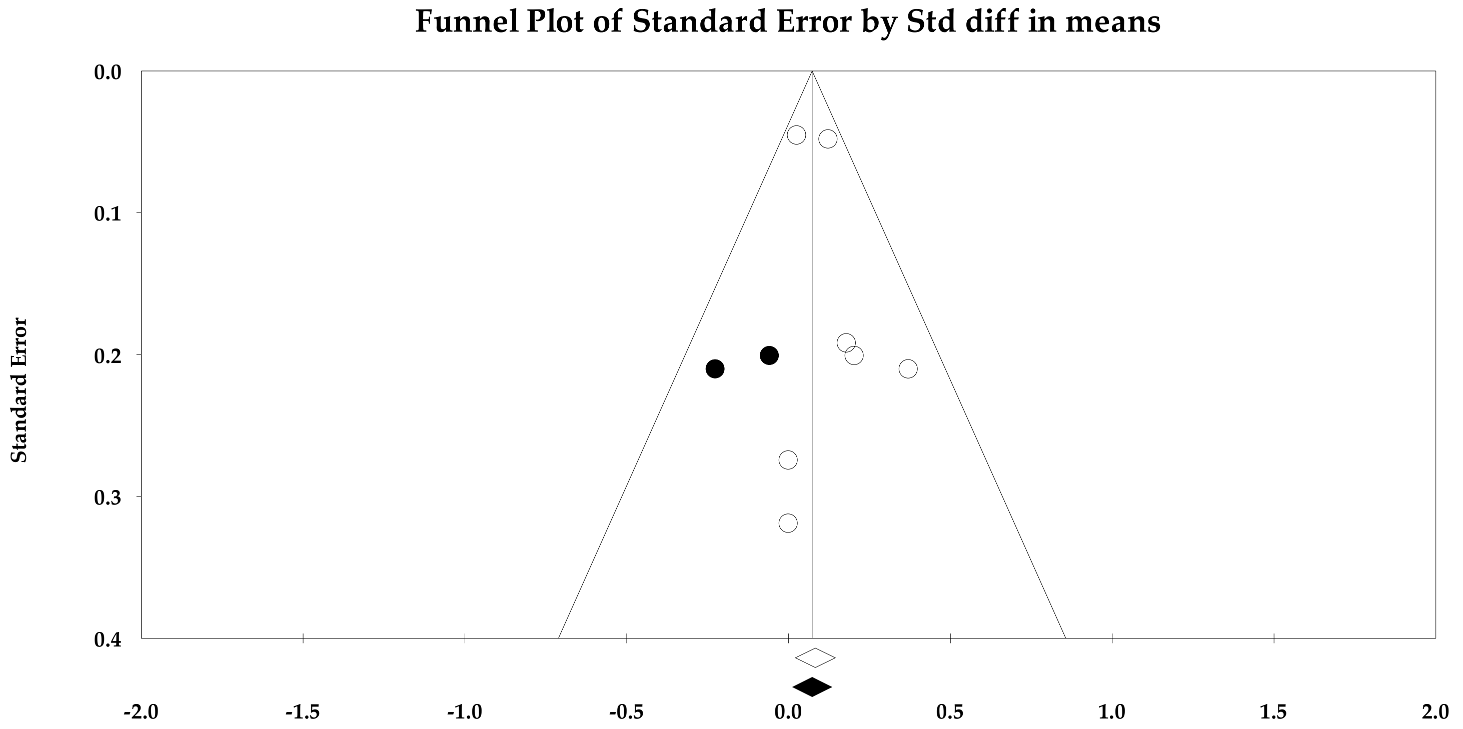

Supplemental Figure S3. Task orientation and performance random effects plots trimmed and filled (*d* samples). The open circles are the data points, and the filled circles are the result of the trim and fill analysis. The clear rhombus is the mean effect size, and the filled rhombus is the trim and filled mean effect size.

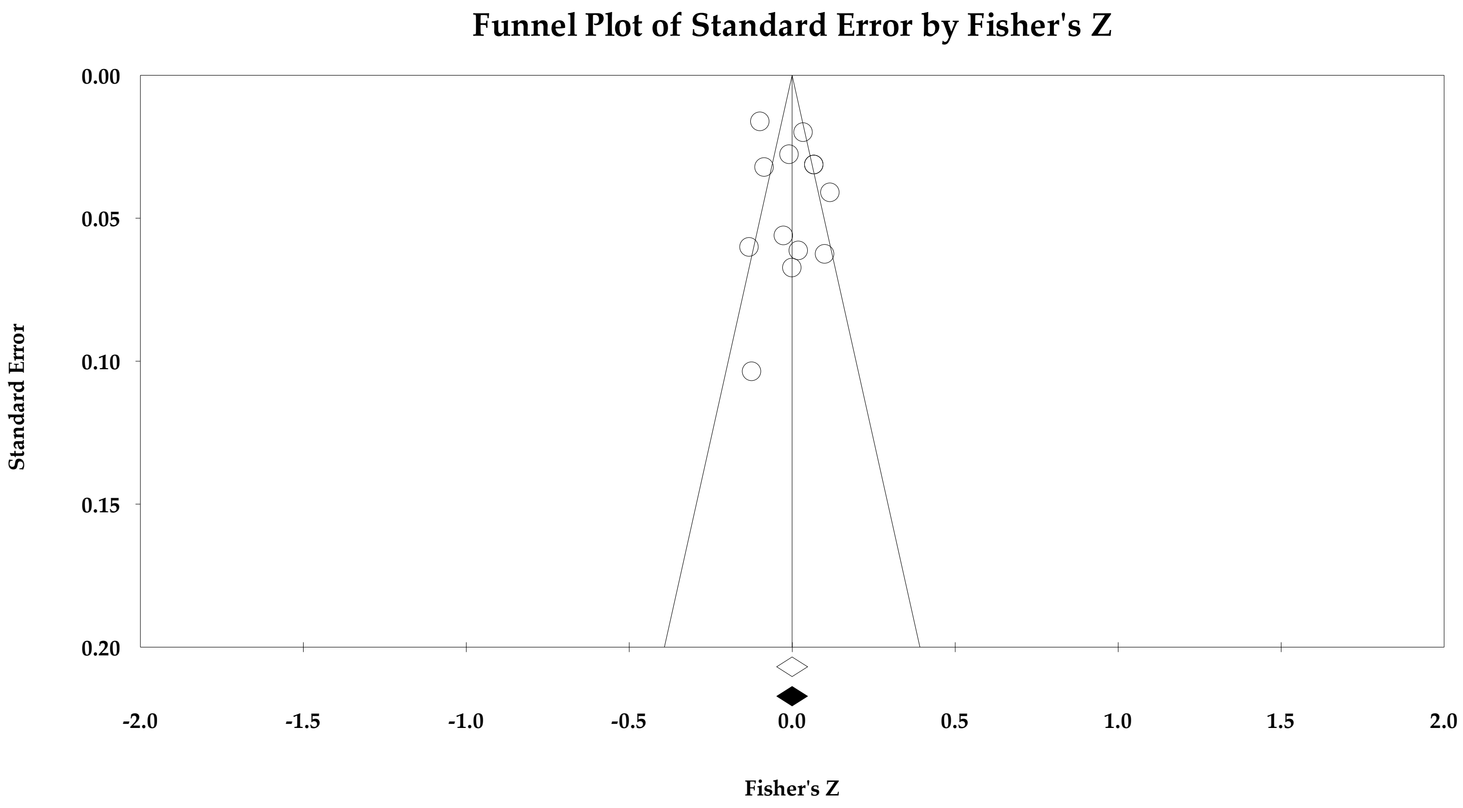

Supplemental Figure S4. Ego climate and performance random effects plots trimmed and filled. The open circles are the data points, and the filled circles are the result of the trim and fill analysis. The clear rhombus is the mean effect size, and the filled rhombus is the trim and filled mean effect size.

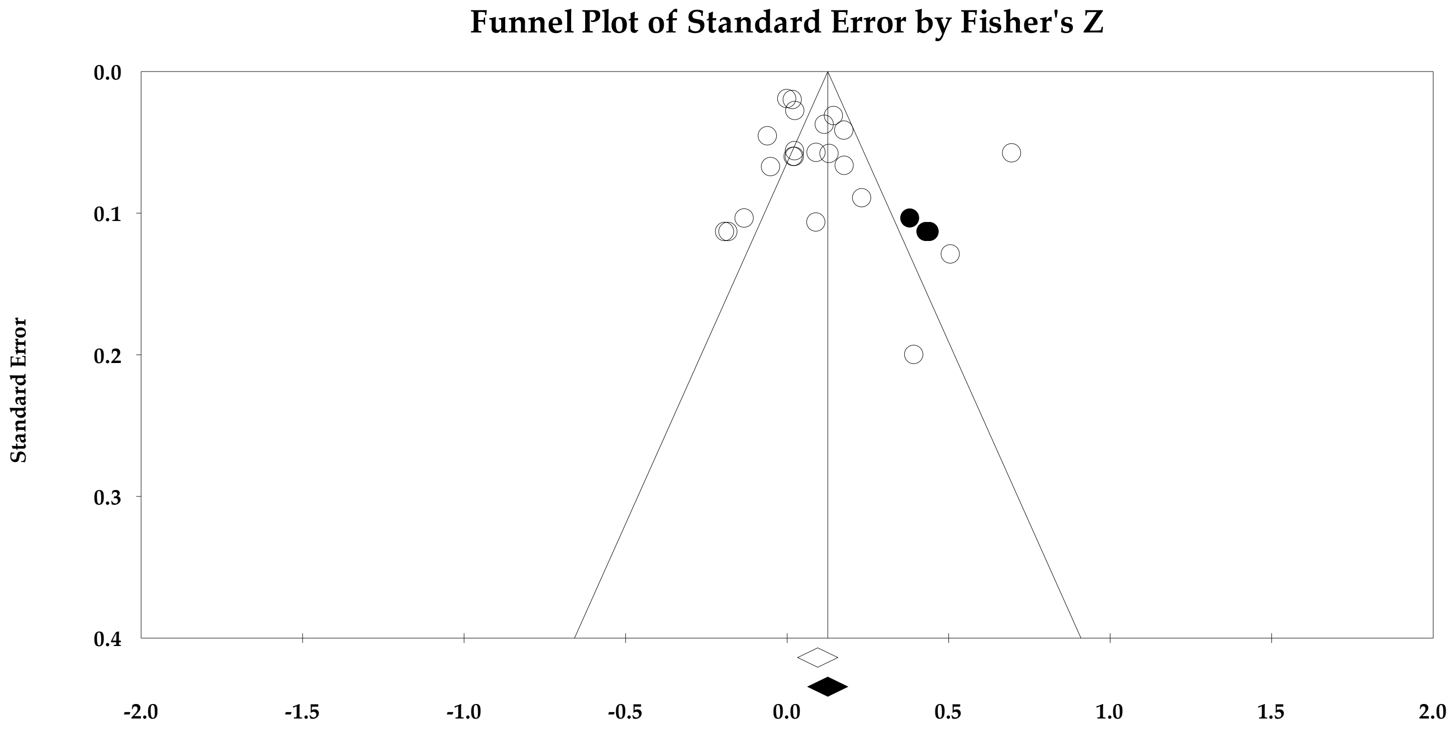

Supplemental Figure S5. Ego orientation and performance random effects plots trimmed and filled (*r* samples). The open circles are the data points, and the filled circles are the result of the trim and fill analysis. The clear rhombus is the mean effect size, and the filled rhombus is the trim and filled mean effect size.

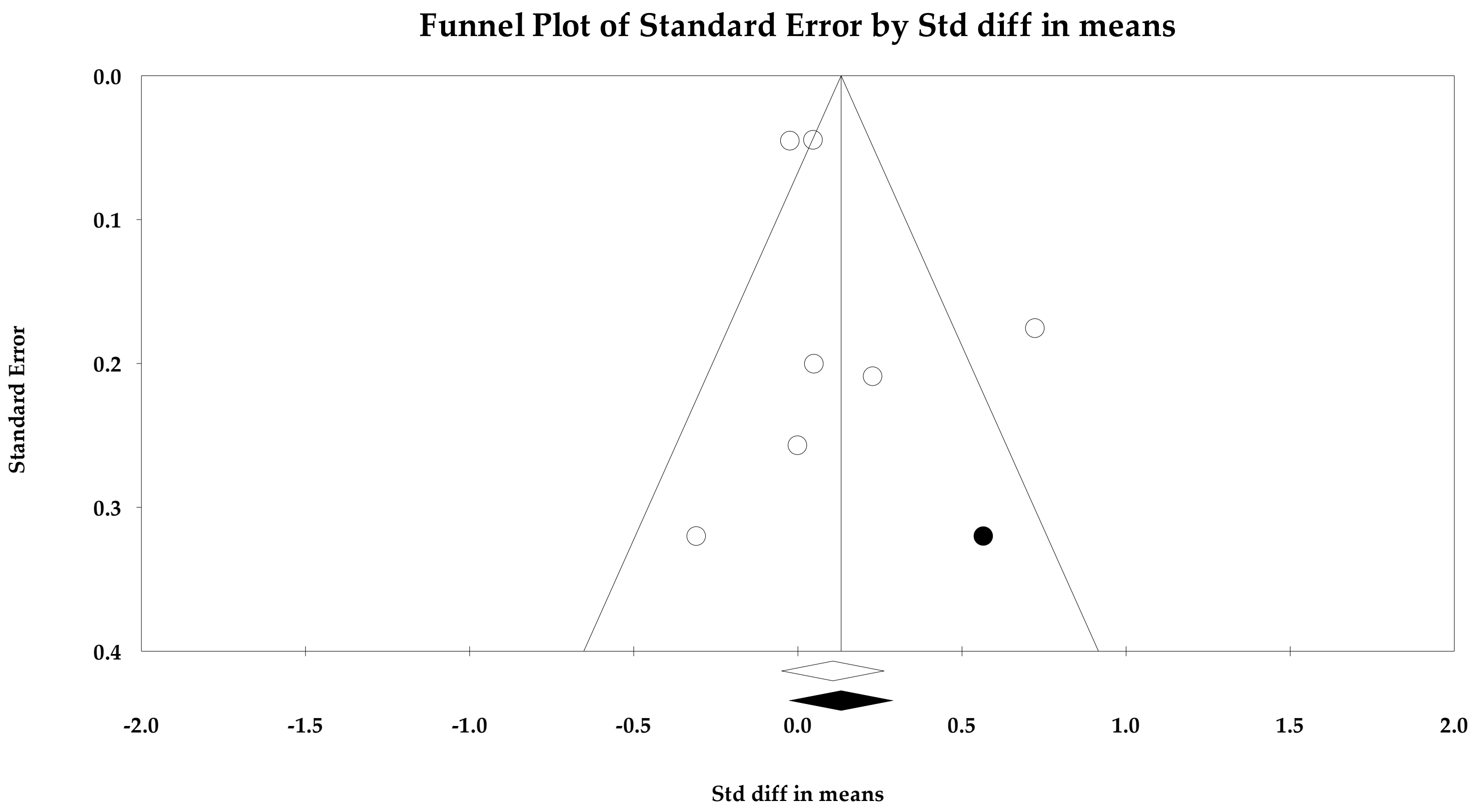

Supplemental Figure S6. Ego orientation and performance random effects plots trimmed and filled (standard difference in means samples). The open circles are the data points, and the filled circles are the result of the trim and fill analysis. The clear rhombus is the mean effect size, and the filled rhombus is the trim and filled mean effect size.

Supplement Figures S7–S12. Remove-one study figures.

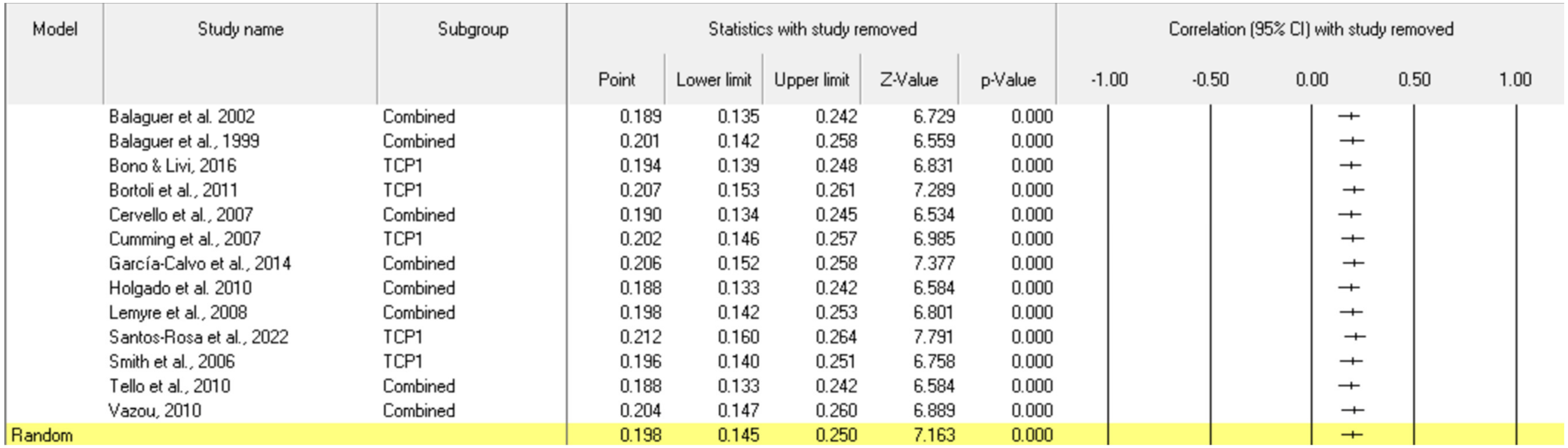

Model	Study name	Subgroup	Statistics with study removed					Correlation (95% CI) with study removed
			Point	Lower limit	Upper limit	Z-Value	p-Value	-1.00 -0.50 0.00 0.50 1.00
	Balaguer et al. 2002	Combined	0.189	0.135	0.242	6.729	0.000	
	Balaguer et al., 1999	Combined	0.201	0.142	0.258	6.559	0.000	
	Bono & Livi, 2016	TCP1	0.194	0.139	0.248	6.831	0.000	
	Bortoli et al., 2011	TCP1	0.207	0.153	0.261	7.289	0.000	
	Cervello et al., 2007	Combined	0.190	0.134	0.245	6.534	0.000	
	Cumming et al., 2007	TCP1	0.202	0.146	0.257	6.985	0.000	
	García-Calvo et al., 2014	Combined	0.206	0.152	0.258	7.377	0.000	
	Holgado et al. 2010	Combined	0.188	0.133	0.242	6.584	0.000	
	Lemyre et al., 2008	Combined	0.198	0.142	0.253	6.801	0.000	
	Santos-Rosa et al., 2022	TCP1	0.212	0.160	0.264	7.791	0.000	
	Smith et al., 2006	TCP1	0.196	0.140	0.251	6.758	0.000	
	Tello et al., 2010	Combined	0.188	0.133	0.242	6.584	0.000	
	Vazou, 2010	Combined	0.204	0.147	0.260	6.889	0.000	
Random			0.198	0.145	0.250	7.163	0.000	

Supplemental Figure S7. Random-effects model statistics (correlation) and forest plots for task climate and performance, with one study removed. Figure references [50–53,56,57,63,64,70,72–74,78].

Model	Study name	Subgroup	Statistics with study removed				
			Point	Lower limit	Upper limit	Z-Value	p-Value
	Alvarez et al., 2018	Combined	0.179	0.115	0.241	5.460	0.000
	Balaguer et al. 2002	Combined	0.178	0.108	0.245	4.981	0.000
	Balaguer et al., 1999	Combined	0.178	0.112	0.243	5.211	0.000
	Bono & Livi, 2016	TOP1	0.171	0.108	0.232	5.302	0.000
	Bortoli et al., 2011	TOP1	0.174	0.110	0.236	5.306	0.000
	Boyd & Callaghan 1994	TOP1	0.180	0.118	0.241	5.603	0.000
	Branco et al., 2024	Combined	0.173	0.109	0.236	5.235	0.000
	Cervello et al., 2007	Combined	0.176	0.112	0.238	5.360	0.000
	Dewar & Kavussanu 2012	Combined	0.175	0.110	0.238	5.235	0.000
	Elferink-Gemser et al., 2015	TOP1	0.175	0.113	0.236	5.450	0.000
	Farkhondeh & Moghaddam 2015	TOP1	0.179	0.116	0.240	5.520	0.000
	Höner & Feichtinger 2016 ob1a	TOP6	0.185	0.129	0.240	6.388	0.000
	Jeong, 2023	TOP1	0.150	0.099	0.201	5.695	0.000
	Knoblochova et al., 2021	TOP1	0.178	0.116	0.240	5.515	0.000
	Lemyre et al., 2008	Combined	0.174	0.110	0.236	5.315	0.000
	Peng & Zhang, 2021 S1	TOP1	0.178	0.116	0.239	5.536	0.000
	Peng & Zhang, 2021 S2	TOP1	0.175	0.112	0.236	5.415	0.000
	Smith et al., 2006	TOP1	0.169	0.106	0.230	5.234	0.000
	Tello et al., 2010	Combined	0.164	0.106	0.221	5.506	0.000
	Tenenbaum et al., 1999	TOP1	0.170	0.108	0.230	5.341	0.000
	van de Pol & Kavussanu, 2011	Combined	0.172	0.109	0.233	5.279	0.000
	Van-Yperen & Duda 1999	Combined	0.174	0.111	0.236	5.328	0.000
	Vazou, 2010	TOP1	0.177	0.113	0.239	5.349	0.000
Random			0.174	0.113	0.234	5.519	0.000

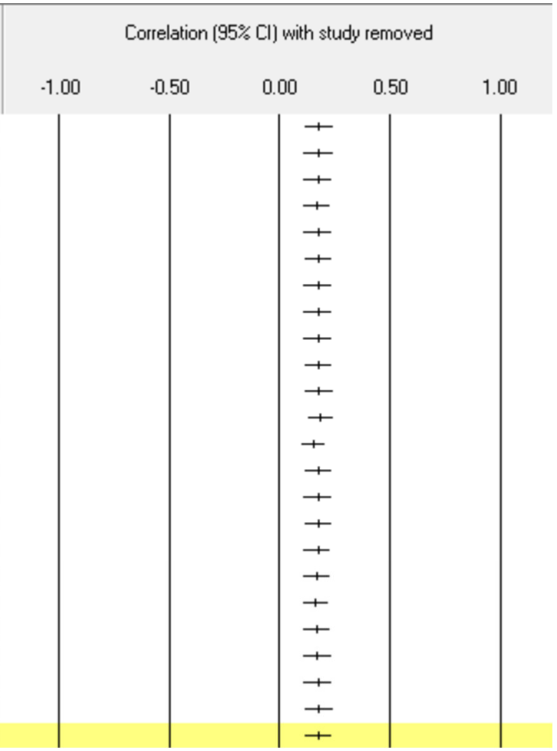

Supplemental Figure S8. Random-effects model statistics (correlation) and forest plots for task orientation and performance, with one study removed. Figure references [49–56,58,60,61,65,67–71,73–78].

Model	Study name	Subgroup	Statistics with study removed						
			Point	Standard error	Variance	Lower limit	Upper limit	Z-Value	p-Value
	e Silva et al. 2010	TOP1	0.080	0.032	0.001	0.018	0.142	2.510	0.012
	Figueiredo et al. 2009 s1	TOP1	0.083	0.032	0.001	0.022	0.145	2.642	0.008
	Figueiredo et al. 2009 s2	TOP1	0.084	0.032	0.001	0.022	0.146	2.647	0.008
	Höner & Feichtinger 2016 ob1b	Combined	0.135	0.044	0.002	0.050	0.220	3.098	0.002
	Höner & Feichtinger 2016 ob2	TOP2	0.053	0.041	0.002	-0.028	0.134	1.278	0.201
	Huijgen et al. 2012	TOP1	0.080	0.032	0.001	0.017	0.142	2.501	0.012
	Kim, 2021	TOP1	0.076	0.032	0.001	0.014	0.138	2.392	0.017
Random			0.083	0.031	0.001	0.021	0.144	2.629	0.009

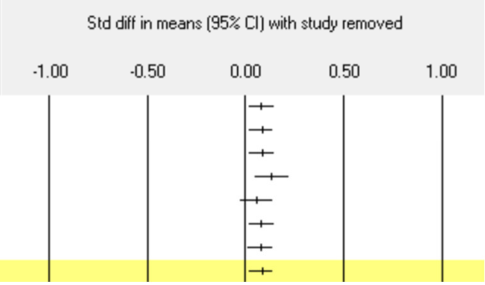

Supplemental Figure S9. Random-effects model statistics (Std diff in means) and forest plots for task orientation and performance, with one study removed. Figure references [59,62,65,66,68].

Model	Study name	Subgroup	Statistics with study removed				
			Point	Lower limit	Upper limit	Z-Value	p-Value
	Balaguer et al. 2002	Combined	-0.004	-0.057	0.048	-0.163	0.871
	Balaguer et al., 1999	Combined	0.000	-0.053	0.053	0.007	0.994
	Bono & Livi, 2016	ECP1	0.004	-0.044	0.053	0.170	0.865
	Bortoli et al., 2011	ECP1	0.001	-0.049	0.051	0.055	0.956
	Cervello et al., 2007	Combined	-0.011	-0.058	0.037	-0.443	0.658
	Cumming et al., 2007	ECP1	-0.002	-0.052	0.048	-0.073	0.942
	García-Calvo et al., 2014	Combined	0.013	-0.027	0.053	0.629	0.529
	Holgado et al. 2010	Combined	-0.007	-0.057	0.042	-0.283	0.777
	Lemyre et al., 2008	Combined	0.009	-0.040	0.057	0.346	0.730
	Santos-Rosa et al., 2022	ECP1	-0.007	-0.056	0.042	-0.284	0.776
	Smith et al., 2006	ECP1	-0.000	-0.050	0.049	-0.019	0.985
	Tello et al., 2010	Combined	-0.007	-0.057	0.042	-0.283	0.777
	Vazou, 2010	Combined	0.008	-0.042	0.058	0.307	0.758
Random			-0.000	-0.048	0.047	-0.017	0.987

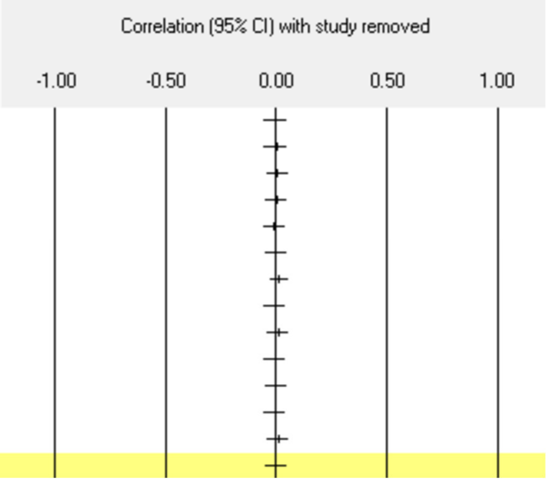

Supplemental Figure S10. Random-effects model statistics (correlation) and forest plots for ego climate and performance, with one study removed. Figure references [50–53,56,57,63,64,70,72–74,78].

Model	Study name	Subgroup	Statistics with study removed				
			Point	Lower limit	Upper limit	Z-Value	p-Value
	Alvarez et al., 2018	Combined	0.095	0.030	0.158	2.871	0.004
	Balaguer et al. 2002	Combined	0.099	0.030	0.168	2.798	0.005
	Balaguer et al., 1999	Combined	0.099	0.031	0.165	2.874	0.004
	Bono & Livi, 2016	EOP1	0.103	0.040	0.165	3.192	0.001
	Bortoli et al., 2011	EOP1	0.098	0.034	0.162	2.975	0.003
	Boyd & Callaghan 1994	EOP1	0.095	0.031	0.157	2.915	0.004
	Branco et al., 2024	Combined	0.090	0.026	0.153	2.735	0.006
	Cervello et al., 2007	Combined	0.093	0.028	0.156	2.816	0.005
	Dewar & Kavussanu 2012	Combined	0.093	0.028	0.158	2.787	0.005
	Elferink-Gemser et al., 2015	EOP1	0.082	0.020	0.143	2.604	0.009
	Höner & Feichtinger 2016 ob1a	EOP6	0.100	0.032	0.168	2.857	0.004
	Jeong, 2023	EOP1	0.061	0.019	0.103	2.822	0.005
	Knoblochova et al., 2021	EOP1	0.089	0.025	0.151	2.743	0.006
	Lemyre et al., 2008	Combined	0.098	0.034	0.162	2.986	0.003
	Peng & Zhang, 2021 S1	EOP1	0.104	0.041	0.166	3.241	0.001
	Peng & Zhang, 2021 S2	EOP1	0.104	0.042	0.166	3.255	0.001
	Smith et al., 2006	EOP1	0.101	0.038	0.164	3.110	0.002
	Tello et al., 2010	Combined	0.092	0.026	0.156	2.734	0.006
	Tenenbaum et al., 1999	EOP1	0.089	0.027	0.151	2.794	0.005
	van de Pol & Kavussanu, 2011	Combined	0.090	0.026	0.153	2.770	0.006
	Van-Yperen & Duda 1999	Combined	0.098	0.034	0.162	2.979	0.003
	Vazou, 2010	EOP1	0.103	0.039	0.166	3.144	0.002
Random			0.094	0.032	0.156	2.979	0.003

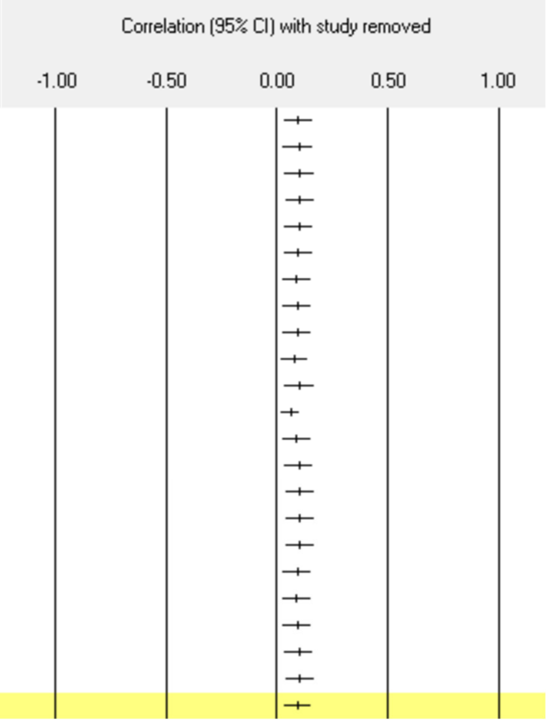

Supplemental Figure S11. Random-effects model statistics (correlation) and forest plots for ego orientation and performance, with one study removed. Figure references [49–56,58,60,65,67,69–71,73–78].

Model	Study name	Subgroup	Statistics with study removed							Std diff in means (95% CI) with study removed
			Point	Standard error	Variance	Lower limit	Upper limit	Z-Value	p-Value	-1.00 -0.50 0.00 0.50 1.00
	e Silva et al. 2010	EOP1	0.015	0.031	0.001	-0.045	0.076	0.491	0.624	
	Figueiredo et al. 2009 s1	EOP1	0.129	0.081	0.007	-0.030	0.288	1.587	0.112	
	Figueiredo et al. 2009 s2	EOP1	0.118	0.085	0.007	-0.049	0.284	1.384	0.166	
	Höner & Feichtinger 2016 ob1b	Combined	0.157	0.127	0.016	-0.093	0.407	1.233	0.218	
	Höner & Feichtinger 2016 ob2	EOP1	0.137	0.138	0.019	-0.134	0.408	0.993	0.321	
	Huijgen et al. 2012	EOP1	0.116	0.087	0.008	-0.055	0.287	1.334	0.182	
	Kim, 2021	EOP1	0.095	0.085	0.007	-0.072	0.261	1.114	0.265	
Random			0.107	0.079	0.006	-0.048	0.261	1.350	0.177	

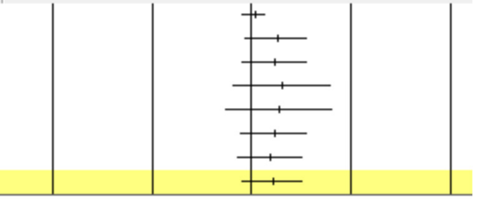

Supplemental Figure S12. Random-effects model statistics (Std diff in means) and forest plots for ego orientation and performance, with one study removed. Figure references [59,62,65,66,68].
